# The impact of a coordinated joint multidisciplinary breast cancer clinic

**DOI:** 10.3332/ecancer.2017.741

**Published:** 2017-06-02

**Authors:** Jamal Zekri

**Affiliations:** College of Medicine, Al Faisal University, PO Box 50927, Riyadh 11533, Saudi Arabia and; King Faisal Specialist Hospital and Research Centre, PO Box 40047, Jeddah 21499, Saudi Arabia

**Keywords:** multidisciplinary, breast cancer, clinic, coordinator

## Abstract

**Introduction:**

Timely access to cancer treatment is expected to improve patients’ satisfaction and treatment outcome. A joint multidisciplinary breast cancer clinic (JMDBCC) was developed in January 2011 which aimed to accelerate access to breast cancer care. Here, we assess the efficacy of this approach.

**Methods:**

Metric data of access to care in 2010 represent the pre-JMDBCC era while those during the subsequent 5 years (2011–2015 inclusive) represent the post-JMDBCC era. The JMDBCC is comprised of three separate but closely adjacent subclinics conducted at the same time representing the three main relevant clinic-based disciplines supported by a breast cancer coordinator. The primary aim of the clinic is to provide service to new patients within 7 days at each of the following stages: acceptance to first clinic visit (S1), first clinic visit to completion of appropriate investigations (S2), and completion of investigations to start of active treatment (S3). Thus, the total duration from acceptance to treatment (S1-3) is aimed to be within 21 days.

**Results:**

Five hundred and fifty visits to the relevant clinics were recorded at the pre-JMDBCC era. Mean time metrics for new patients were as follows: 13, 18, 21, and 46 days for S1, S2, S3, and S1-3, respectively. The JMDBCC achieved reduction in all time metrics from the first year of implementation, reaching 3.6, 4.9, 7.3, and 15.9 days for S1, S2, S3, and S1-3, respectively, in year 2015. The number of new patients and total recorded clinic visits increased from 49/550, respectively, in the pre-JMDBCC era to 92/654, 183/1816, 158/2797, 174/4426, and 180/5883 in subsequent years.

**Conclusions:**

A JMDBCC dramatically accelerates access to specialist multidisciplinary care. All institutions managing patients with breast cancer are encouraged to adopt such a coordinated service. The impact of an effective JMDBCC on disease-specific outcome should be addressed in future studies.

## Introduction

Breast cancer (BC) is the most frequently diagnosed cancer among women worldwide [1]. Certainly, the number of diagnosed cases is rising annually in some parts of the world including Saudi Arabia [2]. Multidisciplinary care (MDC) is a team approach to the provision of healthcare by all relevant medical and allied health disciplines. MDC is accepted as a standard approach to manage patients with cancer including that of the breast. Introduction of BC MDC care is associated with improved survival and reduced variation in outcome among hospitals [3]. A tumour site-specific MD team meeting (MDTM) is a structured venue where relevant specialists discuss and recommend management of individual patients. The MDTM is recommended as an indispensable key component of MDC of patients with cancer [4]. Although MDTMs will promote interdisciplinary communication, but eventually patients will still be reviewed by relevant specialist in different clinics. This will result in multiple clinic visits and time and resource wastage. In addition, patients and specialists will miss the opportunity of hands on live multidisciplinary approach in the presence of the patient when indicated. A joint multidisciplinary breast cancer clinic (JMDBCC) is likely to overcome these challenges. A number of institutions have implemented these clinics. However, the impact on waiting times, resource management, and clinic capacity accommodation is not well reported. A JMDBCC was developed at the author’s institution in January 2011 aiming to accelerate access to breast cancer care. This study was undertaken to evaluate the clinic in reference to its intended goal, namely (a) waiting times metrics and (b) clinic capacity.

## Methods

Our hospital contains 382 regular admission beds excluding emergency, day case, and post-operative recovery beds. It is a public tertiary care health facility for a large catchment area covering all of the Western region of Saudi Arabia, representing about a third of the geographic area of the country. Some hospitals in this area provide comprehensive cancer services (including those for BC). Others provide only partial or no specialist cancer services and thus rely on referral to other hospitals. Appropriate cancer referrals are accepted by the relevant discipline for initial workup and subsequent discussion at cancer site-specific MDTMs. The average number of new cancer cases accepted every year by our hospital during the study period (2010–2015) was 909 cases (range: 841–956).

**Figure 1.**
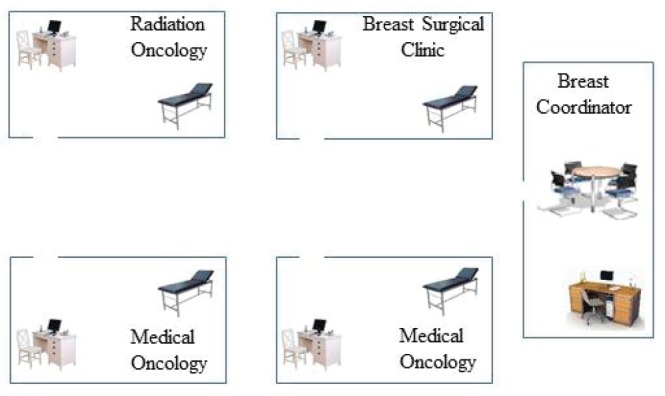
Schematic illustration of the joint multidisciplinary breast cancer clinic.

BC-specific weekly MDTM is already an established component of the service for about 12 years. However, patients were referred to relevant disciplines in different clinics on different days prior to the development of the JMDBCC. Metric data of access to BC care services throughout the year 2010 represent the pre-JMDBCC era while those during the subsequent 5 years (2011–2015 inclusive) represent the post-JMDBCC era. The JMDBCC is comprised of three separate but closely adjacent subclinics conducted at the same time, representing the three main relevant clinic-based disciplines, namely breast surgery, medical oncology, and radiation oncology. The breast surgical subclinic is operated by a qualified consultant breast surgeon with background training in the United Kingdom. The radiation oncology subclinic is operated by a qualified consultant with background training in Canada. Three medical oncologists with specialist interest in BC and background training in the United Kingdom and the United States of America rotate in a structured and organised schedule to operate two medical oncology subclinics in the JMDBCC (Figure 1). The clinic was operating as above only once a week on Tuesday morning. An additional breast surgeon joined the team in February 2015 leading to the implementation of twice a week JMDBBC. The additional service took place weekly on Thursday morning and is operated by one consultant each representing the three main disciplines.

Under direct supervision of the consultants, additional medical staff (residents, higher trainees and assistants) may support different subclinics based on availability, schedule, and interest.

A BC coordinator facilitates the navigation of patients between the three subclinics from the dates of patient’s acceptance. In addition, the coordinator facilitates timely booking of investigations and clinic visits. Each patient is booked and seen in the relevant subclinic by the corresponding specialist. If indicated, the patient is referred to another subclinic immediately. In this case, the referring and the receiving specialists will review the patient jointly and will provide an agreed joint plan of management that is discussed with the patient.

The aim of the clinic is to provide service to new patients within 7 days at each of the following stages: acceptance to first clinic visit (S1), first clinic visit to completion of appropriate investigations (S2), and completion of investigations to start of active treatment (S3). Thus, the total duration from acceptance to treatment (S1-3) is aimed to be within 21 days.

The endpoint of this evaluation is to compare the pre-JMDBCC with the post-JMDBCC in terms of the following: (a) Waiting times metrics: measured by the mean number of days of each stage (S1, S2 & S3) and of the combined three stages (S1-3) for all new patients who attended the JMDBCC clinic in the study period. (b) Clinic capacity: measured by total number of visits (new and follow-up ‘FU’ patients) accommodated in the clinics.

The clinic’s data are routinely collected in Excel software on a quarterly basis since implementation by the BC coordinator as part of her usual duties. Simple mathematics and arithmetic mean were used to analyse the data.

## Results

### Endpoint A: Waiting times metrics for new patients

**Figure 2.**
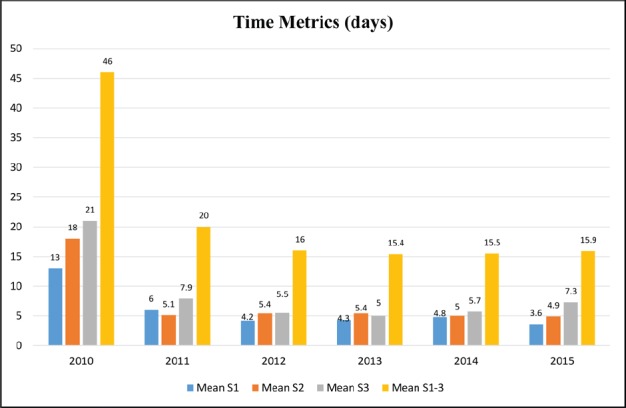
Waiting times metrics for new patients in year 2010 (pre-JMDBCC) and in years 2011-2015 (post-JMDBCC).

Forty-nine new patients were seen in the year 2010 representing the pre-JMDBCC era. Mean time metrics for new patients were as follows: 13, 18, and 21 for S1, S2, and S3, respectively, for these patients. The corresponding metrics in the subsequent years (2011–2015) representing the post-JMDBCC were all within the desired target (except S3 in 2011 = 7.9 and S3 in 2015 = 7.3 days). S1-3 reduced dramatically from 46 days in the year 2010 to less than 21 years in each of subsequent years. Data are depicted in Figure 2.

### Endpoint B: Clinic capacity

**Figure 3.**
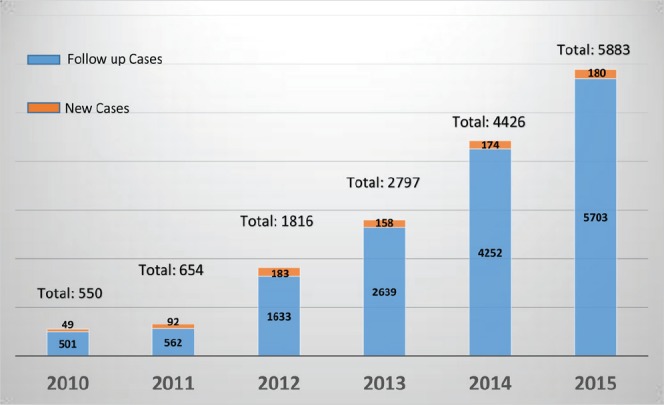
Number of new, follow up and total cases seen in year 2010 (pre-JMDBCC) and in years 2011–2015 (post-JMDBCC).

A total of 550 visits (new: 49 & FU: 501) to the relevant clinics were recorded at the pre-JMDBCC era. The number of visits moderately increased to 654 visits (new: 92 & FU: 562) in the first year of post-JMDBCC era. This was followed by excessive increase in subsequent years reaching 5883 visits (new: 180 & FU: 5703) in year 2015. The data are depicted in Figure 3.

There are two medical oncology subclinics on Tuesdays but only one on the additional Thursday service that started in February 2015.

## Discussion

Many developed countries have introduced different measures to enhance the delivery of cancer services. For example, the United Kingdom National Health Service Cancer Plan (2000) set maximum targets for waiting, including 14 days between general practitioners referral and first hospital appointment, and 31 days between diagnosis and start of treatment [5]. Our JMDBCC dramatically accelerates access to specialist multidisciplinary care with time to starting treatment of 15–16 days despite significant increase in referrals and work load.

Referrals of new patients with diagnosis of breast cancer are accepted to be managed at our hospital. Cases with localised disease are accepted by breast surgeons and those with metastatic disease are accepted by medical oncologists. Historically and prior to the implementation of the JMDBCC, newly accepted patients are first seen in the breast surgical or the medical oncology clinics depending on the accepting specialist. This was followed by planning appropriate treatment and/or referral to other relevant specialist clinics that were scheduled on different days. Despite mandatory discussion in breast MDTM, this practice was expected to potentially (a) Create excessive unnecessary interdisciplinary referrals that consume valuable outpatient clinic slots (b) Increase waiting time to see appropriate specialists (c) Deprive patients and specialists from benefits of real live multidisciplinary review in the presence of the patient. Thus, a JMDBCC was designed and implemented in aim to provide new and existing patients a timely and coordinated access to a real-time multidisciplinary service. Our hospital is a tertiary referral centre, and thus, all most all cases have to have a histological diagnosis of cancer before acceptance. This leaves the BC team with 31 days to start treatment as per the recommendations of the United Kingdom National Health Service Cancer Plan [5]. After deliberation and assessment of available supporting resources, our team felt that it was realistically possible to achieve a more ambitious time of 21 days which was divided between three stages.

The results of our practice audit confirmed that the JMDBCC has achieved this aim. The successful achievement was maintained on the long-term and over the whole 5 years audit period. Standards of care were not compromised despite the increase in total activities of the clinic as indicated by increase in number of new patients and total clinic visits. Management of new patients included (not limited to) the following: (a) Initial first clinic consultation by the appropriate discipline as discussed earlier; (b) Requesting radiological investigations as appropriate such as mammogram, ultrasound, wire localisation, and/or magnetic resonance imaging; (c) Confirmation of histological diagnosis by the institution’s pathology department or occasionally repeating the biopsy if necessary; (d) Discussion at the MDTM and setting treatment options to be discussed with the patient.

Treatment is in line with recognised standards and depends on disease stage at presentation and on other tumour- and patient-related factors. Initial treatment of non-metastatic disease may include: mastectomy with or without reconstruction, breast conservative surgery, and pre-operative chemotherapy. Pre-operative hormonal therapy is rarely recommended at our hospital. Initial treatment of metastatic disease is mostly systemic in the form of chemotherapy or hormonal therapy. Detailed discussion of standards of care and of subsequent therapies is beyond the scope of this report.

The concept of a JMDBCC is practiced at many major cancer centres. However, there is little published confirmation of its advantages. Probably, the earliest reported experience that measured some of the valuable aspects of the JMDBCC was in the year 1994 [6]. Investigators compared patient satisfaction and time metrics of the first 177 patients seen during the first year of the JMDBCC to those of the traditional sequential consultation clinics. Authors reported an improvement in patient satisfaction and reduction in time between diagnosis and the initiation of treatment (42.2 days vs. 29.6 days). We report a similar trend as the mean time from acceptance until start of treatment (S1-3) decreased from 46 days in the pre-JMDBCC to 20 days in the first year of post-JMDBCC era (Figure 2). Interestingly, further reduction in S1-3 stage to 15–16 days was consistently achieved in subsequent years indicating the feasibility and sustainability of the practice (Figure 2). In addition, the JMDBCC capacity registered and exponential growth in the number of accommodated patients indicating its advantageous role in resources management (Figure 3). This growth was achieved with the same number of clinical staff in years 2011–2014 and with only one additional breast surgeon who joined the team in February 2015. Relevant healthcare professionals in the city and the wider catchment area became aware of the existence of the JMDBCC at our institution. This generated an increase in the number of referred cases to our tertiary care hospital which in turn was translated to an increase (87.8%: 92 vs. 49 cases) in the number of accepted new cases in the first year. However, the peak of this increment (273.5%: 183 vs. 49 cases) was reached within two years of implementation (Figure 3) causing significant work load and pressure on the clinic personnel and other supporting services. Members of the MDT implemented a series of coordinated actions aiming at prioritising services to patients with breast cancer in order to maintain the clinic’s achievements. These actions included (a) priority services in pathology, radiology, and operating room facilities, (b) reserving a number of mammography, ultrasound, and magnetic resonance imaging slots, (c) modification of the coordinator’s role to include facilitating timely booking of investigations, (d) contracting another breast surgeon who was successfully employed in February 2015, and (e) contracting a second breast coordination who commenced her role in early 2016 (i.e., after the study period).

We did not investigate the effect of shorter waiting times on patient satisfaction. However, a positive effect has recently been reported by a group from Canada [7]. A group from Salt Lake City (USA) reported high patient satisfaction with the implementation of a JMDBCC. However, they did not comment on waiting times or capacity metrics [8].

It seems that some large cancer centres have only recently implemented the JMDBCC model. In May 2013, the BC team introduced multiteam (MT) clinics at the University of Texas MD Anderson Cancer Center. The results of the first-year experience were presented at the American Society of Clinical Oncology (ASCO) Breast Cancer Meeting. They also reported improvement clinical metrics leading to efficiency and timeliness of access [9].

The achievements of the JMDBCC reflect a collective effort of a well-structured and coordinated MDT including specialist physicians, clinic nurses, clinic clerks, breast cancer coordinator and others. The effect of navigation programmes in adult patients undergoing active anticancer treatments on outcome, such as quality of life, is not confirmed. However, a meta-analysis of four studies with a total of 667 participants concluded that they significantly improved patient satisfaction [10].

Our coordinator has an oncology nursing background. She facilitates timely investigations and clinics/treatments bookings. Her nursing skills and the specialist breast cancer knowledge she acquired through the early era of JMDBCC enabled her to extend her role beyond simple coordination and navigation. She increasingly became involved in providing information and support to patients in the clinic and through telephone communication. It is reasonable to suggest that the coordinator played a major role in achieving the JMDBCC targets at our hospital. This suggestion is supported by findings from the Dana-Farber Cancer Institute, Boston. They reported a significant reduction in time to mastectomy/reconstruction for women with breast cancer through the implementation of the breast coordinator [11].

## Conclusions

A well-structured and supported JMDBCC dramatically accelerates access to breast cancer specialist multidisciplinary care. It also accommodates more patients and thus allows the best use of available resources. All institutions managing patients with breast cancer are encouraged to adopt such a service. The impact of an effective JMDBCC on disease-specific outcome (progression-free and overall survival) should be addressed in future studies.
